# No Difference Between Open and Arthroscopic ATFL Repair, Both Yielding Clinically Significant Improvement in Chronic Ankle Instability: A Randomized Controlled Trial

**DOI:** 10.1177/23259671261417357

**Published:** 2026-04-08

**Authors:** Gwendolyn Vuurberg, Priscilla A. Maria, Rayan Baalbaki, Daniel Haverkamp, Inger N. Sierevelt, Sjoerd A.S. Stufkens, Helder Pereira, Gino M.M.J. Kerkhoffs, the CAISR Trial Study Group, N.A. Altink, C.J.A. van Bergen, L. Blankevoort, X. Crevoisier, C.N. van Dijk, Daniel Hoornenborg, R. Krips, P.A. de Leeuw, J.C. Peerbooms, M.L. Reilingh, D. Sousa, K. Stanekova

**Affiliations:** Department of Orthopaedic Surgery and Sports Medicine, Amsterdam UMC Location University of Amsterdam, Amsterdam, The Netherlands; Department of Orthopaedic Surgery, Amphia Hospital, Breda, the Netherlands; Department of Orthopaedic Surgery and Sports Medicine, Erasmus University Medical Centre–Sophia Children’s Hospital, Rotterdam, the Netherlands; Department of Orthopaedic Surgery and Sports Medicine, Amsterdam UMC location University of Amsterdam, Amsterdam, The Netherlands; Amsterdam Movement Sciences, Aging & Vitality and Sports, Amsterdam, The Netherlands; (Department of Orthopaedic Surgery and Traumatology, CHUV Centre Hospitalier, Universitaire Vaudois, Lausanne, Switzerland; Department of Orthopaedic Surgery and Sports Medicine, Amsterdam UMC Location University of Amsterdam, msterdam, The Netherlands; Xpert Clinics Orthopaedics, Amsterdam, the Netherlands; Department of Orthopaedic Surgery, Flevoziekenhuis Almere, Utrecht, the Netherlands; Department of Orthopaedic Surgery, Flevoziekenhuis Almere, Utrecht, the Netherlands; Department of Orthopaedic Surgery, Albert Schweitzer Hospital, Dordrecht/Zwijndrecht; Department of Orthopaedic Surgery, Albert Schweitzer Hospital, Dordrecht/Zwijndrechtl Department of Orthopaedic Surgery, St. Antonius Hospital, Utrecht, the Netherlands; Orthopaedic Department, Centro Hospitalar Póvoa de Varzim–Vila do Conde, Poóvoa de Varzim, Portugal; Department of Orthopaedic Surgery and Traumatology, CHUV Centre Hospitalier, Universitaire Vaudois, Lausanne, Switzerland; † Department of Orthopaedic Surgery and Sports Medicine, Amsterdam UMC location University of Amsterdam, Amsterdam, The Netherlands; ‡ Department of Radiology and Nuclear Medicine, Rijnstate Hospital, Arnhem, The Netherlands; § Department of Orthopaedic Surgery and Traumatology, CHUV Centre Hospitalier, Universitaire Vaudois, Lausanne, Switzerland; ‖ Montchoisi Clinic, Swiss Medical Network, Lausanne, Switzerland; ¶ Xpert Clinics Orthopaedics, Amsterdam, The Netherlands; # Department of Orthopaedic Surgery, Spaarnegasthuis, Hoofddorp, The Netherlands; ** Amsterdam Movement Sciences, Aging & Vitality and Sports, Amsterdam, The Netherlands; †† Academic Centre for Evidence-Based Sports Medicine (ACES), Amsterdam, The Netherlands; ‡‡ Amsterdam Collaboration on Health & Safety in Sports (ACHSS), IOC Research Centre, Amsterdam, The Netherlands; §§ Life and Health Sciences Research Institute (ICVS), School of Medicine, University of Minho, Braga, Portugal; ‖‖ Orthopaedic Department, Centro Hospitalar Póvoa de Varzim–Vila do Conde, Póvoa de Varzim, Portugal; ¶¶ Amsterdam Movement Sciences, Musculoskeletal Health and Sports, Amsterdam, The Netherlands; ## Members of the CAISR Trial Study Group are listed at the end of the article; Investigation performed at the Amsterdam University Medical Centre (UMC), Albert Schweitzer Hospital Dordrecht, Amphia Hospital Breda, and Xpert Clinics Amsterdam, Netherlands, Service d'orthopédie et traumatologie CHUV Lausanne, Switzerland, and Centro Hospitalar Póvoa de Varzim – Vila do Conde, Portugal

**Keywords:** ankle instability, ATFL injury, inversion sprain, chronic instability, ATFL repair

## Abstract

**Background:**

Lateral ligament repair is a reliable surgical approach for the treatment of chronic ankle instability (CAI). Although the open technique has shown success over time, technological advancements have enabled the increasing use of arthroscopic techniques, with the expectation of improving results. The objective of the current trial was to compare the results of open and arthroscopic anterior talofibular ligament (ATFL) repair in patients with CAI.

**Hypothesis:**

Arthroscopic anatomic repair would lead to superior functional outcome as measured by the Foot and Ankle Outcome Score (FAOS) compared with open anatomic repair.

**Study Design:**

Randomized controlled trial; Level of evidence, 2.

**Methods:**

Patients at 6 participating sites were included if they were age 16 years or older, had a history of ankle inversion sprain, had symptoms ≥6 months, and had mechanical ankle instability. Randomization was performed over open and arthroscopic ATFL repair, with or without retinacular augmentation. Both procedures were performed using all-suture anchors and corresponding instrumentation. The primary outcome measure was functional outcome as measured by the FAOS at 24 months of follow-up.

**Results:**

A total of 41 patients were included: 21 patients in the open repair group and 20 patients in the arthroscopic repair group. The mean ± SD age of the patients at the time of surgery was 32 ± 13 years. No statistically significant difference was found between treatment groups for any of the FAOS subscales at any of the follow-up intervals. Both groups showed statistically significant score improvements across all FAOS subscales (*P* < .005). Complications were reported in 4 patients (18%) in the open surgery group (infection, swelling, deep venous thrombosis, adhesion) and 1 patient (5%) in the arthroscopy group (neurogenic pain) (*P* = .343). In both groups, 1 resprain was reported (5%).

**Conclusion:**

Open and arthroscopic ATFL repairs equally achieved statistically significant improvement in functional outcome and effectively addressed functional and mechanical ankle instability.

**Registration:**

ClinicalTrials.gov database (reference NCT02998333) and the Dutch trial registry (NL55707.018.16).

Ankle inversion sprain is the most frequently encountered traumatic musculoskeletal injury of the lower extremities, affecting both athletes and nonathletes.^12,31^ Lateral ankle sprain (LAS) comprises 80% of all ankle sprains, with an overall incidence of 15% to 20% of all sports injuries.^12,56^ Despite appropriate nonoperative treatment, up to 40% of patients develop chronic ankle instability (CAI), symptoms of which include prolonged periods of pain, immobility, and injury recurrence.^3,39,56^

Understanding etiological factors requires knowledge of the anatomic stabilizing structures of the ankle. The anterior talofibular ligament (ATFL) and calcaneofibular ligament (CFL) are the major static lateral ligamentous stabilizers that are affected in patients with CAI.^7,26,50^ In case of LAS, forced plantarflexion results in decreased stability of the talus within the mortise, explaining the increased load on ligaments. The ATFL is the most commonly injured ligament and therefore the main objective for surgical stabilization.^13,26^ When considering surgical treatment, providers must clinically differentiate between functional and mechanical instability.^23^ Mechanical instability reveals pathologic tibiotalar hypermobility and is associated with reports of giving way.^41^ Functional instability, however, is characterized by subjective reports of instability based on insufficiency in proprioception and neuromuscular control.^23,59^

Prevention and adequate treatment of CAI are of great importance. Repetitive episodes of ankle sprains not only make patients hesitant to maintain an active lifestyle^14^ but also increase the risk of sequelae such as osteochondral lesion of the talus (OLT) due to repeated impaction of the talus onto the tibial plafond, ankle impingement, loose bodies, medial ligament injury, or tendinopathy, as reported in >50% of patients.^25,46,52,60^ This gave rise to the statement that “there is no such thing as a simple ankle sprain,”^29,53^ and both the possibility of a “domino effect” and the beginning of a “silent cascade,” possibly leading to overall joint degeneration,^9^ need to be considered. The high incidence of CAI in combination with the risk of secondary sequelae and concomitant socioeconomic impact emphasizes the need for adequate diagnosis and subsequent treatment.^40,39,46^

If instability persists, despite ≥6 months of nonoperative treatment, surgical treatment may be considered. Surgical repair or reconstruction should be anatomic to obtain the best biomechanical and clinical results. Nonanatomic reconstructions such as tenodesis provided inferior results and have, therefore, been abandoned.^2,6,17,32,33,38,39^ The shift toward anatomic repair revealed a high incidence of concomitant injury of the CFL, which can be explained by the shared anatomic footprint and the common role as an ankle stabilizer. However, historical evidence has shown that solitary ATFL repair provides sufficient stabilization. In case additional strengthening of the anatomic repair is needed, the Gould modification is added (also Bröstrom-Gould). This modification refers to reefing the inferior extensor retinaculum (IER) over the ATFL.^7,30,37^

Anatomic repair may be performed as an open or arthroscopic procedure. Although arthroscopic ATFL repair was first described in the 1990s, it initially failed to gain popularity due to its complexity. Several pioneers such as Corte-Real, Nery, Lui, and Vega have pushed the technique and instrumentation forward.^36,43,54^ In a biomechanical cadaveric study, Drakos et al^15^ showed that there was no difference in biomechanical stability between the 2 techniques. Despite open repair being the gold standard, to our best knowledge, evidence comprises only 25 studies, including only one level 2 randomized controlled trial (RCT) on arthroscopic repair with and without IER reinforcement.^21,28^

The purpose of the current trial was to compare the functional outcomes of open versus arthroscopic repair in patients with chronic lateral ankle instability during a 2-year period after treatment. Drawing on published literature, we considered that both open and arthroscopic repairs establish a stabilized ankle. Based on a recent successful series^42^ and its minimally invasive character, we hypothesized that arthroscopic anatomic repair would lead to superior functional outcomes in Foot and Ankle Outcome Score (FAOS) pain and disability compared with open anatomic repair.

## Methods

### Study Design

The current study was an international, nonblinded, multicenter RCT comparing open and arthroscopic ATFL repair. A total of 6 hospitals/clinics participated. In the Netherlands, the selected study sites were the Albert Schweitzer Hospital Dordrecht, Amphia Hospital Breda, Amsterdam University Medical Centre (UMC) location AMC, and Xpert Clinics Amsterdam. Other participants were Centro Hospitalar Póvoa de Varzim–Vila do Conde from Portugal and Service d’Orthopédie et Traumatologie CHUV Lausanne from Switzerland.

Participating orthopaedic surgeons completed either a physical cadaveric training session or an online meeting before performing their first patient inclusion to discuss technicalities of the procedure, procedural details, and optimizing surgical uniformity. A learning curve was minimized by assignment of the procedure to orthopaedic surgeons experienced in both procedures. To ensure unbiased patient information concerning the procedure, an independent expert was appointed per country to answer patients’ questions regarding the study independent of the treating physician.

The study was approved by the local internal review board of each participating site (reference Amsterdam UMC 2016_136#B2016709) and was subsequently registered in the ClinicalTrials.gov database (reference NCT02998333) and the Dutch trial registry (NL55707.018.16). The trial was coordinated by the Department for Orthopedic Surgery and Sports Medicine at the Amsterdam UMC in Amsterdam, the Netherlands.

### Patient Selection

All patients visiting the outpatient clinic between January 2016 and October 2023 eligible for surgical ATFL repair were screened for meeting the selection criteria. Inclusion criteria were (1) minimum age of 16 years, (2) history of an ankle inversion sprain, (3) persistent complaints for ≥6 months despite nonoperative treatment, and (4) a positive anterior drawer test (ADT).^18,44^ Exclusion criteria were (1) a previous foot or ankle fracture, (2) previous foot or ankle surgery, (3) restriction in range of motion (ROM) of >10° compared with the contralateral side, (4) medial or subtalar instability, (5) severe malalignment and deformities (eg, severe flat foot), and (6) the presence of severe concomitant injury (eg, ankle fractures or OLT >2 cm in diameter).^1^

Ankle instability was defined as isolated lateral ankle instability reported as a sensation of instability of the ankle during sports and/or daily activity, possibly concomitant pain, with ≥1 previous episode of an ankle inversion sprain. Complaints had to persist for ≥6 months despite nonoperative treatment, and mechanical ankle instability was confirmed by a positive ADT.

### Sample Size

Based on a minimal clinically important difference (MCID) in mean FAOS of 25 points for the Sports subscale and a reported standard deviation for CAI patients of 23.3, a significant *P* value of .05, and 90% power, the sample size calculation resulted in a total of 19 patients per treatment arm.^48,49^ The sample size calculation was performed by a clinical epidemiologist.

The COVID-19 pandemic negatively affected the enrollment rate of the trial. To avoid loss to follow-up due to the inability of patients to visit the outpatient clinic during the pandemic, a nonsubstantial amendment was approved accepting missing data from the physical examination. A nonsubstantial amendment was chosen because the initially selected per-protocol design would be suitable for both a noninferiority and a superiority research objective. However, because a superiority design was chosen, a per-protocol analysis was also appropriate to meet our requirements, avoiding an unnecessarily considerable number of patients being excluded due to not being able to attend the outpatient clinic in person during the COVID-19 lockdowns between 2020 and 2022.

### Randomization and Blinding

Participants were randomly allocated to open or arthroscopic ATFL repair by means of a computer-based randomization program by the local coordinating researcher. Randomization was performed using ICH-GCP and ISO 27001 certified online data capture software (Castor EDC). Block randomization with variable block sizes was used, and stratification criteria were defined as participating site and the presence of a small OLT (<2 cm). Additional required procedures for concomitant injury were defined perioperatively. Therefore, this was not included as a stratification criterion. Patients were blinded to the allocated treatment until bandage removal postoperatively. To ensure an objective interpretation of results, data were interpreted according to a blinded interpretation scheme.^27^

### Surgical Procedures

#### Open Anatomic AFTL Repair

A curvilinear skin incision was made from the distal fibula to the midpoint between the lateral talus and the anterior process of the calcaneus. The lateral branch of the superficial peroneal nerve was identified and protected throughout the procedure. Through sharp dissection, subsequent cauterization, and release of ligament or remnants, the ATFL footprint was identified (Appendix Figure A1). Vitalization of the footprint was achieved using a rongeur. Anchor placement was performed according to the device instructions. The 2 suture wires were passed through the ATFL and tied together, tensioning the ATFL to its footprint. In case the ADT revealed persisting instability perioperatively, retinacular reinforcement was performed, similar to the arthroscopic technique.

#### Arthroscopic Anatomic ATFL Repair

Standard anteromedial or centromedial and anterolateral portals were used. An accessory lateral portal was made at the fibula tip for anchor placement. The entire course of the ATFL was visualized (Appendix Figure A2) and assessed for ligament tension and tissue quality. The fibular footprint was prepared for anchor placement using a shaver or electrocoagulation until bleeding bone was achieved.^45^ A blunt trocar was used inside a drill guide. A tunnel of 17 mm in length was created using a 1.4-mm drill bit. Maintaining the guide in the same position and direction, the anchor was introduced into the tunnel through the lateral accessory portal. An 18-gauge needle with a No. 3-0 nylon loop was used as a suture passer. The needle went through the proximal half of the ATFL. The nylon loop was retrieved outside the joint, and the solid blue wire of the anchor was passed through the loop and subsequently pulled out through the skin. The ligament was tightened onto the fibula using the regular knot-tying technique. In selected cases, a second anchor was placed just proximal to the footprint of the ATFL using the same technique, suturing the IER over the repaired ATFL.

Ankle stability and ROM were tested and confirmed. For both procedures, the same anchors (all-suture JuggerKnot, with a diameter of 1.4 mm, by Zimmer Biomet) were used.

### Additional Procedures

In patients with CAI, concomitant injury has been reported in up to 77% of cases, with a wide range of pathologies encountered perioperatively.^8^ To mirror clinical practice, when perioperative concomitant injury necessitating additional procedures (eg, removal of impingement) was encountered, these were performed according to standard protocol, also to level the playing field for postoperative recovery.

### Postoperative Management

A conventional nonweightbearing plaster of Paris splint was applied in a plantigrade position for the first 2 weeks. Stitches were removed 10 to 14 days after surgery. Patients were allowed to begin active dorsiflexion and plantarflexion, as well as partial weightbearing with a walker boot and crutches, 2 weeks after surgery. Full weightbearing was achieved around 4 to 6 weeks after surgery. Rehabilitation under the supervision of a physical therapist was recommended.

### Outcome Measures

Selected outcome measures included results from physical examination by the treating physician and patient-reported outcome measures (PROMs). All data were pseudonymized and collected using online data capture software (Castor EDC). Physical examination was conducted before surgery, directly after performing surgery, and at 3 and 6 months postoperatively. Questionnaires were collected preoperatively as well as at 3, 6, 12, and 24 months after surgery. There were no changes in outcome measures after commencement.

#### Primary Outcome

The primary study outcome was the mean FAOS improvement compared with baseline for both treatment groups. An MCID was defined as 25 points on the FAOS Sports scale.^49^ The FAOS was assessed using a domain-specific approach, encompassing Pain, Symptoms, Activities of daily Living (ADL), Sports, and Quality of Life, with scores ranging from 0 to 100 points.

#### Secondary Outcomes

Secondary outcomes included pain, disability, and the degree of experienced ankle instability, measured using the Numeric Rating Scale (NRS) and Cumberland Ankle Instability Tool (CAIT). The NRS questions focused on pain and satisfaction, ranging from 0 (no pain/dissatisfied) to 10 points (immense pain/completely satisfied). The CAIT includes 9 items focusing on ankle instability with a score ranging from 0 to 30. For the CAIT, the predefined cutoff was used to differentiate between functional ankle instability (≤11 points) or a stable ankle (≥12 points).^24,57^ The MCID was defined as 25 points per FAOS subscale and 2 points for the total CAIT or NRS.^16,47,57^

Additional secondary outcomes included the degree of mechanical ankle instability during physical examination as measured using the ADT. The ADT results were reported according to the modified anterior talar translation (M-ATT) grading scale (grade 0, 0-2 mm; grade 1, 3-5 mm; grade 2, 6-10 mm; grade 3, 11-15 mm).^11^ ROM was recorded in degrees of dorsiflexion and plantarflexion using a goniometer.

#### Additional Outcomes

Regarding surgery, additional procedures performed were recorded as well as complications and recurrence of ankle instability after surgery.

### Statistical Analysis

Statistical analysis was performed using IBM SPSS Statistics for Windows Version 28.0.1.1. Based on a superiority hypothesis, all outcomes were analyzed according to the intention-to-treat principle.

The PROM scores were compared between and within treatment groups for 2 years of follow-up using mixed model analyses. Results were reported as crude and adjusted mean difference (MD) with 95% CI. Adjustment was performed for age, sex, body mass index (BMI), OLT, and baseline score. PROMs are described as means with standard deviations for each follow-up moment, and additional MDs between treatment groups with 95% CI, were calculated. The ADT was used to report the amount of subluxation in millimeters as categorized using the M-ATT and analyzed using the Wilcoxon signed rank test to evaluate the improvement in mechanical stability and Mann-Whitney *U* test to compare stability between treatment groups. ROM was presented as mean and standard deviation per follow-up interval before and after surgery and compared using the paired *t* test (mean and 95% CI). A *P* value of <.05 was considered statistically significant.

## Results

### Patient Selection

**Figure 1.**
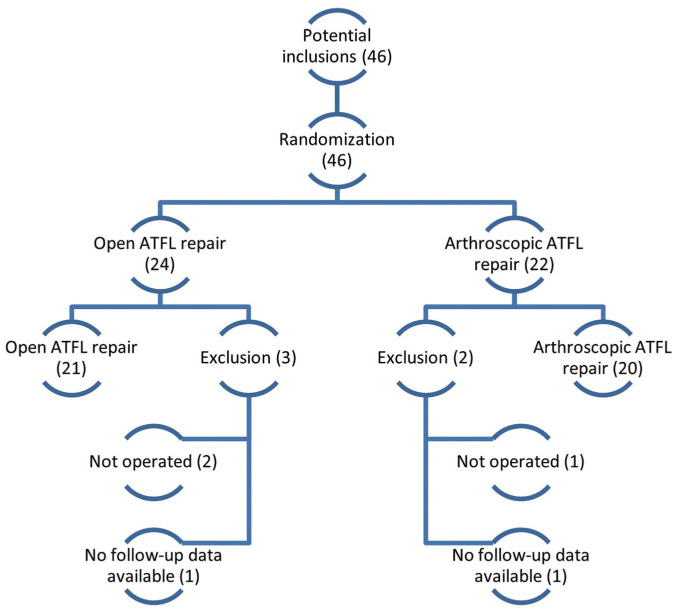
Flowchart of patient enrollment. ATFL, anterior talofibular ligament.

A total of 46 patients were identified between January 2016 and October 2023 as potential candidates for this study, included, and randomized. A total of 5 patients were classified as inclusion failures (Figure 1). The remaining 41 patients underwent surgery according to their assigned randomization code, with 21 patients included in the open treatment arm and 20 patients in the arthroscopic group (Table 1). No patients switched between treatment groups. The study ended February 2025. A total of 22 patients were female (54%), and the right ankle was affected in 24 cases (59%). The mean age of the patients at the time of operation was 32 ± 13 years and the mean BMI was 26.8 ± 4.6 kg/m^2^. An OLT was diagnosed in 5 patients (12%). Of a total of 205 follow-up questionnaires, 22 questionnaires were not filled out during follow-up (11%). Missing questionnaires included preoperative (n = 1), 3 months (n = 7), 6 months (n = 4), 12 months (n = 3), and 24 months (n = 7). Information on physical examination was missing for a total of 6 individual patients due to no-shows at the outpatient clinic (eg, COVID-19 restrictions), including preoperative (n = 1), 3-month (n = 1), and 6-month (n = 4) assessments. One perioperative measurement was missing due to a recording failure.

**Table 1:** Patient Demographic Characteristics per Treatment Arm^*a*^

Demographic Characteristic	Open (n = 21)	Arthroscopic (n = 20)
Sex, male:female, n	11:10	8:12
Age, y	32.9 ± 14.9	30.6 ± 11.7
Body mass index	27.1 ± 5.1	26.5 ± 4.2
Affected ankle, right:left, n	14:7	10:10
OLT present	2 (9.5)	3 (15.0)
Additional procedure	6 (28.6)	7 (30.0)
Resection of anterior impingement	1	4
Bröstrom-Gould including calcaneofibular ligament repair	2	0
Gould augmentation/capsular reefing	1	0
OLT	2	3
Osteophyte/fragment resection, other	2	0
Hospital/clinic	21 (100)	20 (100)
Amphia Hospital	2 (9.5)	5 (25.0)
Albert Schweitzer Hospital	5 (23.8)	4 (20.0)
Amsterdam UMC	3 (14.3)	2 (10.0)
Xpert Clinics	3 (14.3)	4 (20.0)
Centro Hospitalar Póvoa de Varzim	2 (9.5)	1 (5.0)
Service D’orthopédie et Traumatologie	6 (28.6)	4 (20.0)

a Data are expressed as mean SD or n (%) unless otherwise noted. OLT, osteochondral lesion of the talus.

Additional procedures were performed in 12 patients (29%). These included debridement and microfracture in patients with an OLT (n = 5), resection of anterior impingement (AMI) (n = 3), a combination of AMI reduction and Gould augmentation (n = 1), resection of a lateral bony fragment (n = 1), capsular reeving and osteophyte resection (n = 1), and additional repair of the CFL and IER (n = 1).

### Patient-Reported Outcomes

The FAOS revealed no significant score difference comparing open and arthroscopic ATFL repair during 24-month follow-up. Furthermore, no statistically significant differences between groups were found for the NRS and CAIT. Additionally, none of the differences exceeded the predefined MCID when comparing both treatment arms (Table 2).

**Table 2:** Mean Difference During 24 Months of Follow-up (β), Crude and Adjusted for Age, Sex, Body Mass Index, Osteochondral Lesion of the Talus, and Baseline^*a*^

	β_crude_ (95% CI)	*P*	β_adjusted_ (95% CI)	*P*
FAOS				
Pain	4.5 (–3.5 to 12.4)	.265	4.7 (–1.4 to 10.8)	.124
Symptoms	5.9 (–4.2 to 16.1)	.243	4.2 (–2.0 to 10.4)	.175
ADL	3.6 (–3.7 to 10.9)	.322	3.5 (–1.4 to 8.4)	.152
Sports	1.6 (–9.7 to 12.9)	.779	1.9 (–7.0 to 10.7)	.667
QoL	6.6 (–3.4 to 16.6)	.189	6.9 (–2.3 to 16.0)	.137
NRS				
Rest	–0.2 (–0.9 to 0.5)	.618	–0.4 (–0.8 to 0.0)	.071
Walking	–0.7 (–1.5 to 0.2)	.147	–0.5 (–1.2 to 0.2)	.134
Running	0.0 (–1.2 to 1.2)	≥.999	0.0 (–1.0 to 1.1)	.971
Sports	–0.5 (–1.7 to 0.7)	.433	–0.1 (–1.1 to 0.9)	.862
CAIT				
Affected	0.1 (–3.9 to 4.1)	.965	0.1 (–3.5 to 3.6)	.968
Not affected	0.6 (–3.1 to 4.3)	.731	0.5 (–1.6 to 2.7)	.609

a ADL, Activities of Daily Living; CAIT, Cumberland Ankle Instability Tool; FAOS, and Ankle Outcome Score; NRS, Numeric Rating Scale; QoL, Quality of Life.

#### FAOS Values

**Figure 2.**
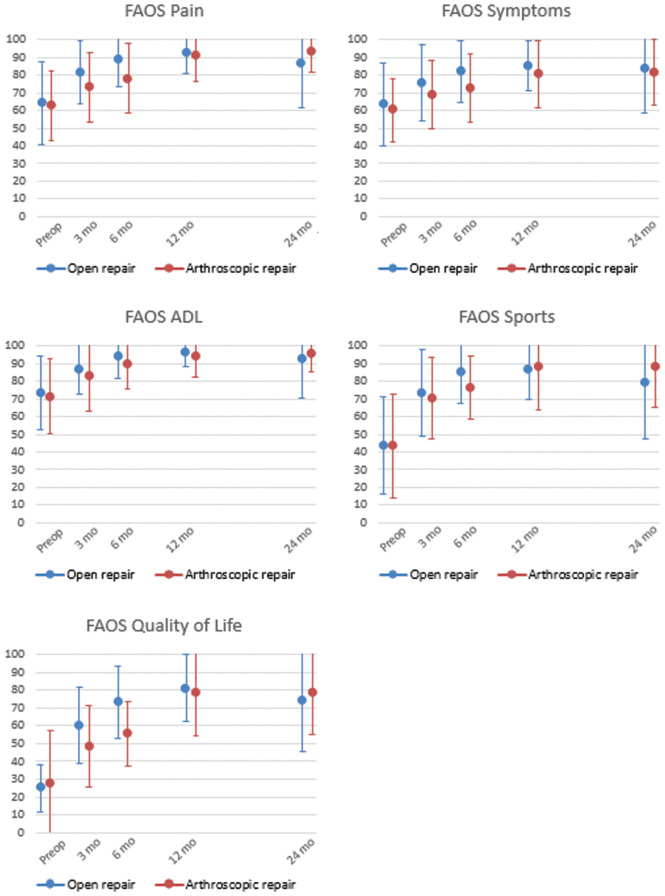
Foot and Ankle Outcome Score (FAOS) (mean ± SD) for open (blue) and arthroscopic (red) repair per subscale indicating overlap of mean and standard deviation, representing that the scores did not differ between treatment arms. ADL, Activities of Daily Living.

All FAOS subscales showed statistically significant improvement over time (*P* < .05), which was not statistically significantly affected by treatment arm or the presence of an OLT (Figure 2). The greatest recovery was seen in the Quality of Life subscale. Mean preoperative FAOS Pain scores improved from 64 ± 23 to 87 ± 25 and from 62 ± 20 to 93 ± 12 for open and arthroscopic repair, respectively. The greatest improvement in pain was appreciated during the first 12 months (MD 29) (Appendix Table A1, available online). Although the recovery between 3 to 6 months was not statistically significant, between 12- and 24-month follow-up statistically significant recovery was seen for all subscales, MD ranging from 13.4 to 36.7.

#### CAIT Score

**Figure 3.**
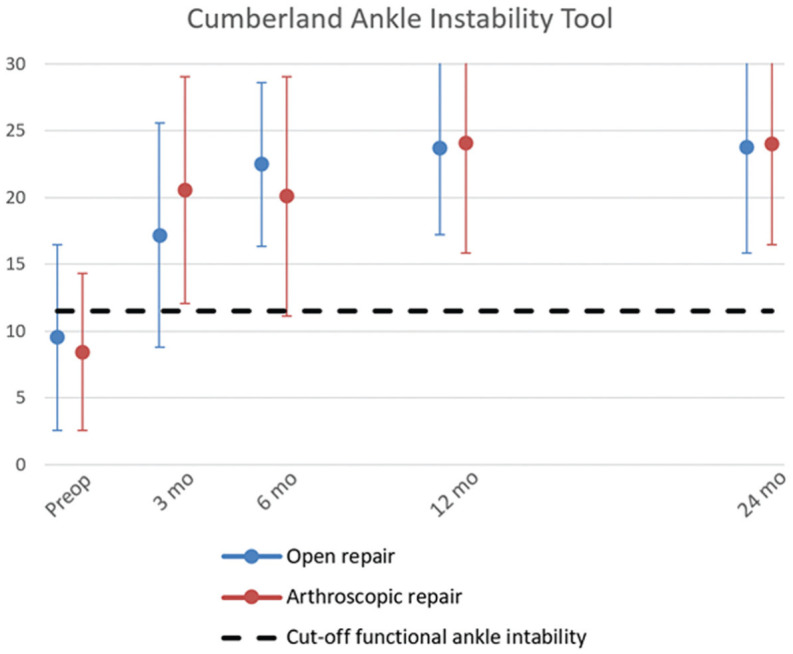
Improvement in functional ankle instability indicated by mean score ± SD per follow-up moment, revealing the average patient reaching functional stability at the 3-month follow-up and all included patients reaching functional stability at the 12-month follow-up.

The overall mean CAIT score significantly improved from 8.4 points (95% CI, 4.8-12.1) to 19.0 (95% CI, 15.9-22.0), 19.8 (95% CI, 15.5-24.2), 20.9 (95% CI, 17.2-24.6), and 24.7 (95% CI, 20.9-28.5) for the 3-, 6-, 12-, and 24-month assessments, respectively (*P* < .05). At the final follow-up, open repair had a mean CAIT score of 23.8 ± 7.9, and arthroscopic repair had a mean score of 24.0 ± 7.5 (Figure 3). Based on the mean scores, functional stability was achieved at the 3-month follow-up for both groups, with scores at the final follow-up indicating functional stability for both the operated and the contralateral ankle. Both groups exceeded the MCID of 2 points after surgical stabilization compared with the preoperative baseline. No statistically significant difference was seen between groups.

#### NRS Pain

**Figure 4.**
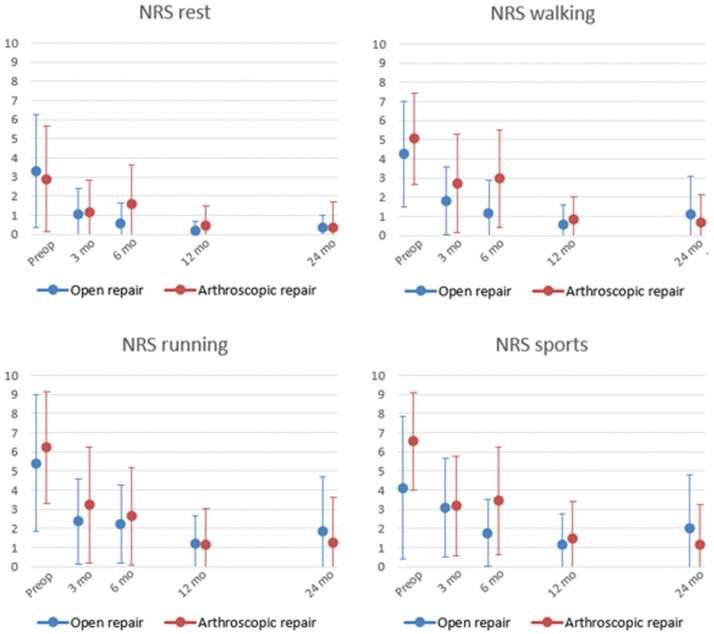
Mean Numeric Rating Scale (NRS) Pain scores per subscale for rest, walking, running, and sports, revealing overall improvement without a significant difference between treatment groups.

Compared with the preoperative scores, follow-up intervals showed statistically significant improvement in pain scores for all pain subscales (*P* < .005 to *P* = .019) (Appendix Table A2, available online). For all pain assessments, the greatest overall improvement was seen in the first 3 months (MD 1-3) (Figure 4). However, when we assessed scores per treatment group, arthroscopy showed significant improvement in NRS Sports at 12 and 24 months, whereas open repair showed improvement at only the 12-month follow-up.

#### Patient Satisfaction

Overall, patients expressed great satisfaction by the end of treatment across all parameters. ADLs showed statistically significant increase in satisfaction of MD 6.9 (95% CI, 6.5-7.3) from 4.3 ± 2.7 to 8.2 ± 2.3 at 24-month follow-up (*P* < .005). Function was appreciated with a mean of 5.4 ± 2.6 points preoperatively and 8.9 ± 1.6 points postoperatively (*P* < .005). Subanalyses revealed a clinically relevant greater satisfaction regarding function at 6 months in the open repair group compared with arthroscopic treatment (MD 3.6 vs 1.5). Overall treatment satisfaction scores increased from a mean of 5.8 ± 2.8 preoperatively to 9.0 ± 1.9 at 24-month follow-up (*P* < .005).

### Physical Examination

#### ADT

Preoperatively, the ADT ranged from 1 to 3, with a median of 3 and 2 in the open and arthroscopic ATFL repair groups, respectively (Appendix Figure A3). Both groups revealed significant improvement in mechanical ankle instability over time to a median ADT score of 0 (*P* < .005) and did not significantly differ between groups per follow-up interval (*P* = .333-920).

#### ROM

The ROM as measured using a goniometer preoperatively, directly after surgery, and at both the 3- and 6-month follow-up points did not reveal any statistically significant difference (*P* = .70). Neither the arthroscopic nor the open ATFL repair procedures significantly changed joint stiffness (arthroscopy group *P* = .985 and *P* = .981 and open group *P* = .578 and *P* = .184 for dorsiflexion and plantarflexion, respectively) (Appendix Table A3, available online).

### Use of Pain Medication

At the start of the study, 9 patients (4 open group, 5 arthroscopic group) reported the use of paracetamol (n = 6), nonsteroidal anti-inflammatory drugs (NSAIDs) (n = 3), and/or tramadol (n = 2) for ankle pain before study initiation. At the 3-month follow-up, 7 patients (3 open group, 4 arthroscopic group) reported the use of pain medications (4 paracetamol; 3 NSAIDs) for their ankle pain. In the open repair group, 1 patient reported the use of NSAIDs at the 6-month follow-up, and 1 patient reported using NSAIDs at both 12- and 24-month follow-ups due to tearing of ligaments of the contralateral ankle. One patient reported using paracetamol, NSAIDs, and tramadol without further explanation.

### Complications

A total of 9 complications were reported, all of which had resolved by the final follow-up. Reported complications included infection, persistent swelling, deep venous thrombosis, adhesion, and resprain in the open treatment group (n = 7; 33%). In the arthroscopic treatment group, neurogenic pain and resprain were reported (n = 2; 10%). No statistically significant difference was found between the treatment arm and the incidence of complications (*P* = .343) or reported resprains (*P* = .473). Despite an even distribution of additional procedures performed between treatment arms, this was not reflected in an even number of complications.

Resprain of the operated ankle was reported in both the open and the arthroscopy groups by 1 patient from each group, respectively (Appendix Table A4, available online). Both patients reported a resprain at the 3-month follow-up. One additional patient reported spraining their contralateral ankle, resulting in rupture of the ATFL. No other adverse effects were reported.

## Discussion

### Main Findings

Technical advancements reducing the complexity of minimally invasive procedures have facilitated the increasing number of arthroscopic ATFL repairs. Due to its minimally invasive character, arthroscopic ATFL repair was hypothesized to provide better functional outcomes. However, the current trial showed no statistically significant difference in FAOS when comparing open and arthroscopic repair. Both groups showed statistically significant and clinically relevant improvement on all FAOS subscales, as well as for the CAIT and NRS. Physical examination revealed improvement in ADT without ROM restriction. Despite some findings exceeding the MCID comparing both treatment arms (eg, CAIT 3- and 6-month follow-up) (Appendix Table A5, available online), PROM scores did not significantly differ between treatment groups. Significant score improvement exceeding the MCID was first achieved by the arthroscopic treatment arm for the NRS Sports subscale at 3 months only. All other outcomes achieved both statistical significant improvement exceeding the MCID at similar time points.

### Published Literature

Previous meta-analyses support excellent results in both treatment groups. However, Zhi et al^62^ found a greater pain reduction in the arthroscopic treatment group, suggesting this was due to its minimally invasive character. Our results supported this finding only for the NRS, specifically focusing on pain during sports. Comparing the 6- to 24-month follow-up revealed a statistically significant and clinically relevant score difference in favor of arthroscopic repair. Guelfi et al^19^ reported improved scores on the foot function index in arthroscopic repair compared with open repair, a superiority our results did not support.

Remarkably, the CAIT score improved for both the operated and the contralateral ankle. This may be explained by the fact that patients experience both mechanical and functional instability. Although the mechanical instability is treated through surgery, functional instability improves through training. These exercises are performed bilaterally, also benefiting the contralateral ankle.^56^ Based on mechanical stability testing (ADT), not all patients reached an optimal score after surgery. Although this may be multifactorial, one of the explanations includes basic anatomy. Because the ATFL and CFL share a common footprint and, thereby, partially common biomechanical properties, this could explain why concomitant CFL injury is often seen in patients with CAI (up to 44%).^34,42^ Recent studies shed light on the existence of connecting fibers, insinuating that joint load-sharing properties between the ATFL and CFL go beyond just a common footprint.^5,10^ Despite a recent RCT reporting good results of isolated ATFL repair, these findings allow debate that even better results may be accomplished by repairing both the ATFL and CFL to optimally restore loading imbalances.^30^

### Clinical Implications

When multiple procedures are available and in the absence of an evidently superior technique regarding clinical outcomes, other parameters should be considered when defining the preferred procedure. Factors that may contribute are economic factors (eg, procedure costs, available surgery equipment), operating time, risk of complications, and recurrence risk. The current RCT shows a 5% resprain rate in both the open repair group and the arthroscopic repair group within 2 years after surgery. Based on the relatively small sample size, it is unknown how this finding compares with the 10% recurrence rate reported in the literature for both techniques.^35^ Overall, sprains are the result of not only ligamentous laxity, which is resolved by ATFL repair, but also loss of proprioception. The latter requires training, which was commenced 2 weeks after surgery in the current trial. In our cohort, it is unknown whether patients who experienced recurrent sprains followed an adequate postoperative training program.

In the literature of both techniques, a wide range of technical variations have been described with an increasing number of publications on minimally invasive surgery. The higher number of publications on arthroscopic repair does not necessarily equal a better technique. In shoulder surgery, a similar trend is seen where technical advancement in arthroscopic treatment showed both increasing complexity in surgical technique (ie, double-row) and an increase in cost and surgery time.^4,51^ It is important to distinguish true advancement from hyperbole and to critically appraise aspects of new surgical techniques, including higher complication rates.^58^ Although postoperative complication rates were as high as 20%, they were lower than the reported complication rate of CAI due to subsequent sequelae (up to 50%).^55^ Additionally, procedural costs may be a topic of concern.^4^ Because arthroscopic ATFL repair is associated with higher costs, it may not be the surgical approach of choice. However, if joint inspection through minimally invasive surgery is required, needle arthroscopy can be considered. Currently, the demand for both elective and emergency medicine is increasing exponentially on a global scale. Technical advancements may facilitate complex procedures but are simultaneously associated with high costs and longer surgery times of surgery.^20,61,62^ Therefore, in the absence of a statistically significant difference in clinical outcomes between both treatment options, it may be debated whether the arthroscopic procedure should be a preferred technique. Together, we are responsible for both today’s and tomorrow’s healthcare.^22^ Sustainability of care, and therefore cost efficiency and affordability, cannot be disregarded.

### Limitations

Summarizing this first RCT comparing open versus arthroscopic ATFL repair reveals statistically significant improvement in all patients without statistically significant effect of treatment arm or the presence of an OLT. The strengths of this RCT are from methodological origin, performing block randomization to ensure equal allocation per participating center, and the participation of 2 surgeons per center performing their preferred procedure to minimize performance bias. Targets of improvement for future comparable studies include a better overview of inclusions, which would have led to earlier inclusion closure with the calculated 19 patients per treatment arm. The uneven distribution of additional procedures for concomitant injury between treatment groups may be considered a limitation, even though this reflects clinical practice. Concomitant injury has been reported in up to 77% of patients with CAI.^8^ An OLT was considered the most important confounder, which did not affect outcomes. Therefore, the other minor additional injuries and procedures were unlikely to have influenced outcomes more than they would in routine clinical workup. Additionally, a more hands-on follow-up would have been preferred. Although postoperative wound checks, including physical examination, were conducted, the COVID-19 pandemic meant that some follow-ups were delayed from the initial target date. Surgical times were not initially recorded and will be a focus for future study. Five patients were excluded despite an intention-to-treat protocol. The reasons for exclusion include not receiving the study treatment (n = 3) and no data available for analysis (n = 2). These inclusions were not expected to affect our analyses and results.

A target for future research could be a cost-effectiveness and sustainability comparison between both procedures to enable a socially responsible choice in the treatment between open or arthroscopic ATFL repair. An individualized patient approach is warranted.

## Conclusion

Open and arthroscopic ATFL repair techniques both accomplished clinically relevant improvement in functional outcome and offered remedy to both functional and mechanical ankle instability. The absence of an evidently superior technique indicates that both treatments were equally able to treat ankle instability. The procedure most suitable for the specific occasion is determined by the orthopaedic surgeon while also taking into consideration patient outcomes and related financial constraints.

## Authors

CAISR Trial Study Group: N.A. Altink, BSc (Department of Orthopaedic Surgery and Sports Medicine, Amsterdam UMC Location University of Amsterdam, Amsterdam, The Netherlands); C.J.A. van Bergen, MD, PhD (Department of Orthopaedic Surgery, Amphia Hospital, Breda, the Netherlands; Department of Orthopaedic Surgery and Sports Medicine, Erasmus University Medical Centre–Sophia Children’s Hospital, Rotterdam, the Netherlands); L. Blankevoort, MD, PhD (Department of Orthopaedic Surgery and Sports Medicine, Amsterdam UMC location University of Amsterdam, Amsterdam, The Netherlands; Amsterdam Movement Sciences, Aging & Vitality and Sports, Amsterdam, The Netherlands); X. Crevoisier (Department of Orthopaedic Surgery and Traumatology, CHUV Centre Hospitalier, Universitaire Vaudois, Lausanne, Switzerland); C.N. van Dijk, MD, PhD (Department of Orthopaedic Surgery and Sports Medicine, Amsterdam UMC Location University of Amsterdam, Amsterdam, The Netherlands); Daniel Hoornenborg, MD (Xpert Clinics Orthopaedics, Amsterdam, the Netherlands); R. Krips, MD, PhD (Department of Orthopaedic Surgery, Flevoziekenhuis Almere, Utrecht, the Netherlands); P.A. de Leeuw, MD, PhD (Department of Orthopaedic Surgery, Flevoziekenhuis Almere, Utrecht, the Netherlands); J.C. Peerbooms, MD, PhD (Department of Orthopaedic Surgery, Albert Schweitzer Hospital, Dordrecht/Zwijndrecht); M.L. Reilingh, MD, PhD (Department of Orthopaedic Surgery, Albert Schweitzer Hospital, Dordrecht/Zwijndrechtl Department of Orthopaedic Surgery, St. Antonius Hospital, Utrecht, the Netherlands); D. Sousa, MD (Orthopaedic Department, Centro Hospitalar Póvoa de Varzim–Vila do Conde, Póvoa de Varzim, Portugal); K. Stanekova (Department of Orthopaedic Surgery and Traumatology, CHUV Centre Hospitalier, Universitaire Vaudois, Lausanne, Switzerland).

## Supplemental Material

sj-docx-1-ojs-10.1177_23259671261417357 – Supplemental material for No Difference Between Open and Arthroscopic ATFL Repair, Both Yielding Clinically Significant Improvement in Chronic Ankle Instability: A Randomized Controlled Trial Supplemental material, sj-docx-1-ojs-10.1177_23259671261417357 for No Difference Between Open and Arthroscopic ATFL Repair, Both Yielding Clinically Significant Improvement in Chronic Ankle Instability: A Randomized Controlled Trial by Gwendolyn Vuurberg, Priscilla A. Maria, Rayan Baalbaki, Daniel Haverkamp, Inger N. Sierevelt, Sjoerd A.S. Stufkens, Helder Pereira, Gino M.M.J. Kerkhoffs, the CAISR Trial Study Group, N.A. Altink, C.J.A. van Bergen, L. Blankevoort, X. Crevoisier, C.N. van Dijk, Daniel Hoornenborg, R. Krips, P.A. de Leeuw, J.C. Peerbooms, M.L. Reilingh, D. Sousa and K. Stanekova in Orthopaedic Journal of Sports Medicine

## Figures and Tables

**Figure S1.**
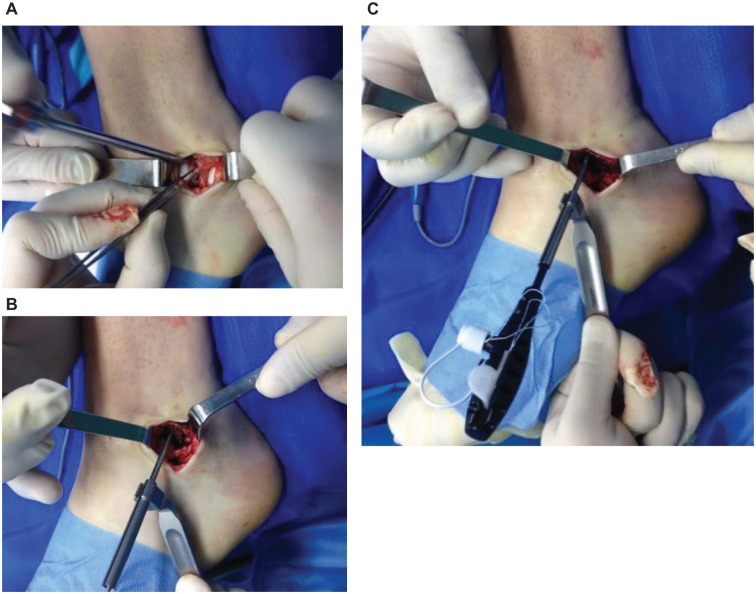
Open ATFL repair: A. identification and preparation of the ATFL footprint, B. drill guide for suture placement, C. suture placement for ATFL repair and tensioning.

**Figure S2.**
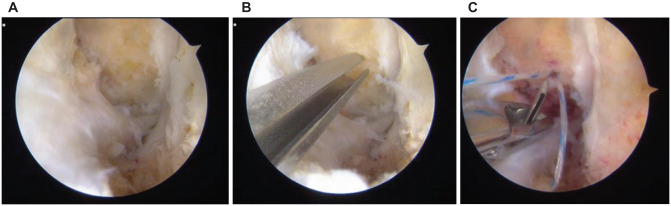
Arthroscopic ATFL repair: A. identification of the ATFL, B. drill guide for suture placement at the ATFL footprint, C. suture retriever for ATFL repair and tensioning of the ligament.

**Figure S3.**
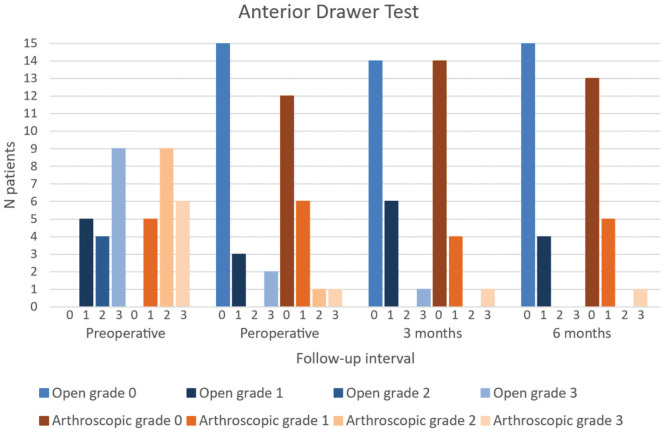
Quantitative overview of improvement in mechanical ankle instability as measured using the ADT per follow-up interval.
