# Supplementary material for: No Difference Between Open and Arthroscopic ATFL Repair, Both Yielding Clinically Significant Improvement in Chronic Ankle Instability: A Randomized Controlled Trial
Source: Orthop J Sports Med. 2026 Apr 8;14(4):23259671261417357. doi: 10.1177/23259671261417357 (PMC13065292; doi:10.1177/23259671261417357)
Supplement: sj-docx-1-ojs-10.1177_23259671261417357 – Supplemental material for No Difference Between Open and Arthroscopic ATFL Repair, Both Yielding Clinically Significant Improvement in Chronic Ankle Instability: A Randomized Controlled Trial [file sj-docx-1-ojs-10.1177_23259671261417357.docx]

# Supplementary material

**Supplementary tables**

Table S1. Mean score improvement (MD) per FAOS sub-scale compared to baseline (preoperative score) per treatment arm and 95% confidence interval. Gray marked cells indicate not statistically significant change compared to baseline.

| **Follow-up interval** | **Treatment arm** | **3 months** | **6 months** | **12 months** | **24 months** |
| --- | --- | --- | --- | --- | --- |
| FAOS pain | Open repair | 18 (-1 – 36) | 25 (7 – 42) | 29 (11 – 46) | 23 (4 – 42) |
|  | Arthroscopic repair | 11 (5 – 26) | 16 (0 – 31) | 29 (13 – 24) | 31 (15 – 46) |
| FAOS symptoms | Open repair | 12 (-8 – 32) | 18 (0 – 37) | 22 (3 – 40) | 20 (0 – 40) |
|  | Arthroscopic repair | 9 (-9 – 26) | 12 (-5 – 29) | 20 (3 – 37) | 21 (3 – 39) |
| FAOS ADL | Open repair | 13 (-3 – 29) | 20 (8 – 38) | 23 (8 – 38) | 19 (3 – 35) |
|  | Arthroscopic repair | 12 (-3 – 37) | 18 (3 – 33) | 23 (8 – 38) | 25 (9 – 40) |
| FAOS sports | Open repair | 30 (6 – 54) | 41 (19 – 63) | 43 (21 – 64) | 36 (13 – 59) |
|  | Arthroscopic repair | 27 (5 – 49) | 33 (11 – 55) | 44 (23 – 67) | 18 (5 – 41) |
| FAOS QoL | Open repair | 35 (15 – 56) | 48 (29 – 67) | 56 (37 – 75) | 49 (29 – 69) |
|  | Arthroscopic repair | 21 (2 – 39) | 28 (10 – 46) | 51 (33 – 70) | 51 (32 – 69) |

Table S2. Mean score improvement (MD) per NRS pain scale compared to baseline (preoperative score) per treatment arm and 95% confidence interval. Gray marked cells indicate no statistically significant change compared to baseline.

| **Follow-up interval** | **Treatment arm** | **3 months** | **6 months** | **12 months** | **24 months** |
| --- | --- | --- | --- | --- | --- |
| NRS rest | Open repair | 2 (1 – 4) | 3 (1 – 4) | 3 (2 – 5) | 3 (1 – 4) |
|  | Arthroscopic repair | 2 (0 – 4) | 1 (0 – 3) | 2 (1 – 4) | 3 (1 – 4) |
| NRS walking | Open repair | 2 (1 – 4) | 3 (1 – 5) | 4 (2 – 5) | 3 (1 – 5) |
|  | Arthroscopic repair | 2 (0 – 4) | 2 (0 – 4) | 4 (2 – 6) | 4 (2 – 6) |
| NRS running | Open repair | 3 (0 – 6) | 3 (1 – 6) | 4 (2 – 7) | 4 (1 – 6) |
|  | Arthroscopic repair | 3 (0 – 6) | 4 (1 – 6) | 5 (3 – 8) | 5 (2 – 8) |
| NRS sport | Open repair | 1 (-2 – 4) | 2 (0 – 5) | 3 (0 – 6) | 2 (-1 – 5) |
|  | Arthroscopic repair | 3 (1 – 6) | 3 (1 – 6) | 5 (3 – 8) | 5 (3 – 8) |

Table S3. Mean range of motion (ROM) per follow-up interval with corresponding standard deviation for the operated/affected ankle.

| **Follow-up interval** | **Open repair**  Mean (95%CI) | | **Arthroscopic repair**  Mean (95%CI) | |
| --- | --- | --- | --- | --- |
|  | **Dorsiflexion** | **Plantarflexion** | **Dorsiflexion** | **Plantarflexion** |
| Preoperative | 11.9 (±5.9) | 45.0 (±10.4) | 9 (±5.2) | 41.5 (±9.7) |
| Directly postoperative | 11.0 (±5.8) | 39.0 (±11.5) | 9.1 (±5.6) | 41.5 (±7.8) |
| 3 months postop | 9.5 (±5.0) | 43.8 (±7.4) | 9.7 (±4.7) | 40.8 (±9.6) |
| 6 months postop | 11.3 (±6.0) | 44.5 (±8.5) | 9.4 (±4.8) | 40.5 (±9.1) |

Table S4. Postoperative complications in percentage of total number of patients and reported re-sprains.

| **Reported complications** | **Open (n = 21)** | **Arthroscopy (n=20)** |
| --- | --- | --- |
| None | 17 (81%) | 19 (95%) |
| Infection | 1 (5%) | - |
| Persistent swelling | 1 (5%) | - |
| Deep venous thrombosis (DVT) | 1 (5%) | - |
| Adhesion due to scar tissue | 1 (5%) | - |
| Neurogenic pain radiating to fourth metatarsal | - | 1 (5%) |
| **Re-sprain** | **Open (n = 21)** | **Arthroscopy (n=20)** |
| Operated ankle | 1 (5%) | 1 (5%) |
| Rupture ATFL   - Operated ankle - Contralateral ankle | 1 (5%)   - - - 1 (5%) | - |

Table S5. Mean PROM scores and corresponding standard deviations of the FAOS, CAIT, and NRS.

| **Follow-up interval** | **Treatment arm** | **Preoperative** | **3 months** | **6 months** | **12 months** | **24 months** |
| --- | --- | --- | --- | --- | --- | --- |
| Foot and Ankle Outcome Score  (MCID 25 points) | | | | | | |
| FAOS pain | Open repair | 64.1 (±23.3) | 81.1 (±18.4) | 89.1 (±15.8) | 92.7 (±11.5) | 87.1 (±25.3) |
|  | Arthroscopic repair | 62.5 (±19.8) | 73.2 (±18.7) | 78.2 (±16.9) | 90.6 (±15.3) | 93.2 (±11.8) |
| FAOS symptoms | Open repair | 63.5 (±23.2) | 74.3 (±22.0) | 81.8 (±17.7) | 85.2 (±14.0) | 83.4 (±24.7) |
|  | Arthroscopic repair | 60.4 (±17.8) | 69.1 (±19.5) | 72.6 (±19.5) | 80.2 (±19.6) | 81.4 (±18.6) |
| FAOS ADL | Open repair | 73.6 (±21.0) | 86.9 (±14.7) | 93.8 (±12.1) | 96.4 (±7.8) | 92.6 (±22.0) |
|  | Arthroscopic repair | 71.3 (±21.2) | 83.1 (±20.2) | 89.5 (±14.2) | 94.0 (±12.3) | 95.8 (±10.7) |
| FAOS sports | Open repair | 43.8 (±27.4) | 73.6 (±24.4) | 84.8 (±17.1) | 86.3 (±16.5) | 79.4 (±31.6) |
|  | Arthroscopic repair | 43.5 (±29.4) | 70.3 (±23.0) | 76.5 (±17.9) | 87.1 (±24.4) | 88.3 (±23.4) |
| FAOS QoL | Open repair | 25.1 (±13.3) | 60.4 (±22.4) | 73.3 (±20.4) | 81.1 (±18.9) | 74.4 (±29.2) |
|  | Arthroscopic repair | 27.7 (±13.6) | 48.3 (±21.1) | 55.4 (±22.9) | 80.8 (±17.4) | 78.6 (±21.5) |
| Cumberland Ankle Instability Tool  (MCID 2 points) | | | | | | |
| CAIT operated ankle | Open repair | 9.8 (±7.2) | 18.2 (±7.9) | 22.5 (±6.3) | 23.7 (±6.6) | 23.8 (±8.2) |
|  | Arthroscopic repair | 8.5 (±6.0) | 20.6 (±8.7) | 20.1 (±9.2) | 23.8 (±8.6) | 24.0 (±7.7) |
| Numeric Rating Scale  (MCID 2 points) | | | | | | |
| NRS rest | Open repair | 3.2 (±3.2) | 0.7 (±0.9) | 0.5 (±0.8) | 0.2 (±0.6) | 0.4 (±0.7) |
|  | Arthroscopic repair | 2.6 (±2.8) | 1.0 (±1.5) | 1.5 (±2.2) | 0.4 (±1.0) | 0.4 (±1.4) |
| NRS walking | Open repair | 4.1 (2.7) | 1.4 (±1.4) | 0.8 (±1.1) | 0.6 (±1.1) | 1.2 (±2.1) |
|  | Arthroscopic repair | 4.9 (±2.6) | 2.6 (±2.3) | 3.0 (±2.7) | 0.8 (±1.2) | 0.7 (±1.5) |
| NRS running | Open repair | 5.1 (±3.6) | 2.4 (±2.3) | 2.2 (±2.1) | 1.2 (±1.6) | 1.9 (±2.9) |
|  | Arthroscopic repair | 6.1 (±3.0) | 2.9 (±2.8) | 2.6 (±2.7) | 1.2 (±1.9) | 1.3 (±2.4) |
| NRS sports | Open repair | 4.4 (±3.8) | 3.1 (±2.6) | 1.9 (±1.8) | 1.3 (±1.7) | 2.1 (±3.0) |
|  | Arthroscopic repair | 6.6 (±2.6) | 3.2 (±2.7) | 3.4 (±2.9) | 1.4 (±2.0) | 1.2 (±2.1) |
| NRS satisfaction ADL | Open repair | 4.2 (±2.6) | 7.1 (±2.6) | 7.2 (±3.0) | 8.1 (±2.3) | 7.6 (±3.0) |
|  | Arthroscopic repair | 4.2 (±2.8) | 6.7 (±2.3) | 6.8 (±1.7) | 8.6 (±1.4) | 8.8 (±1.2) |
| NRS satisfaction function | Open repair | 4.8 (±2.3) | 7.5 (±2.4) | 8.6 (±2.3) | 9.2 (±1.1) | 8.7 (±2.0) |
|  | Arthroscopic repair | 5.7 (±2.8) | 7.1 (±2.1) | 7.3 (±1.8) | 8.9 (±1.2) | 9.1 (±1.0) |
| NRS satisfaction sports | Open repair | 6.2 (±2.5) | 8.2 (±2.5) | 8.7 (±2.3) | 9.1 (±1.2) | 8.6 (±2.5) |
|  | Arthroscopic repair | 5.5 (±3.0) | 8.2 (±1.3) | 8.3 (±1.3) | 9.1 (±1.4) | 9.3 (±1.0) |

**Supplementary figures**


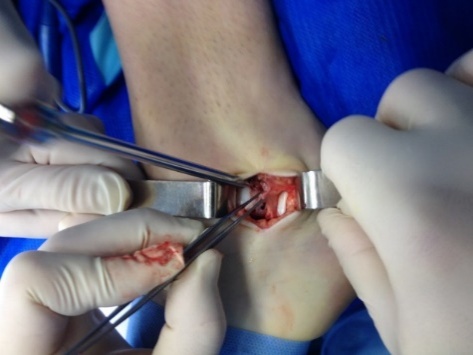


A


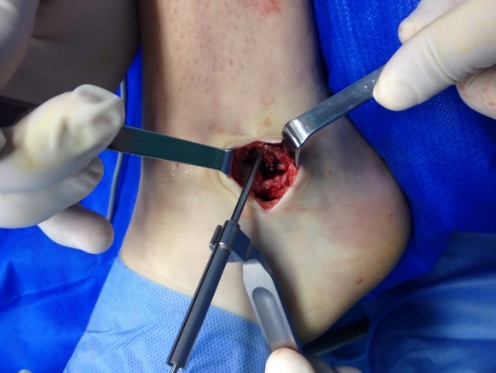


B


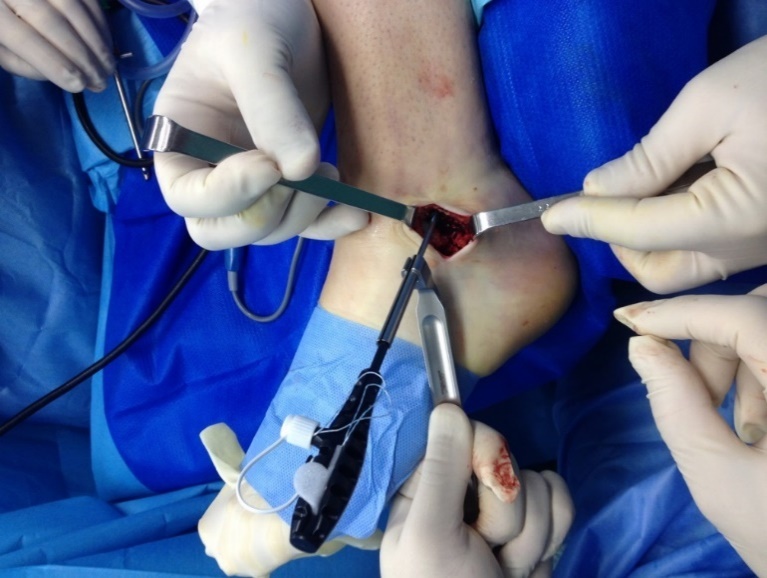


C

Figure S1. Open ATFL repair: A. identification and preparation of the ATFL footprint, B. drill guide for suture placement, C. suture placement for ATFL repair and tensioning.


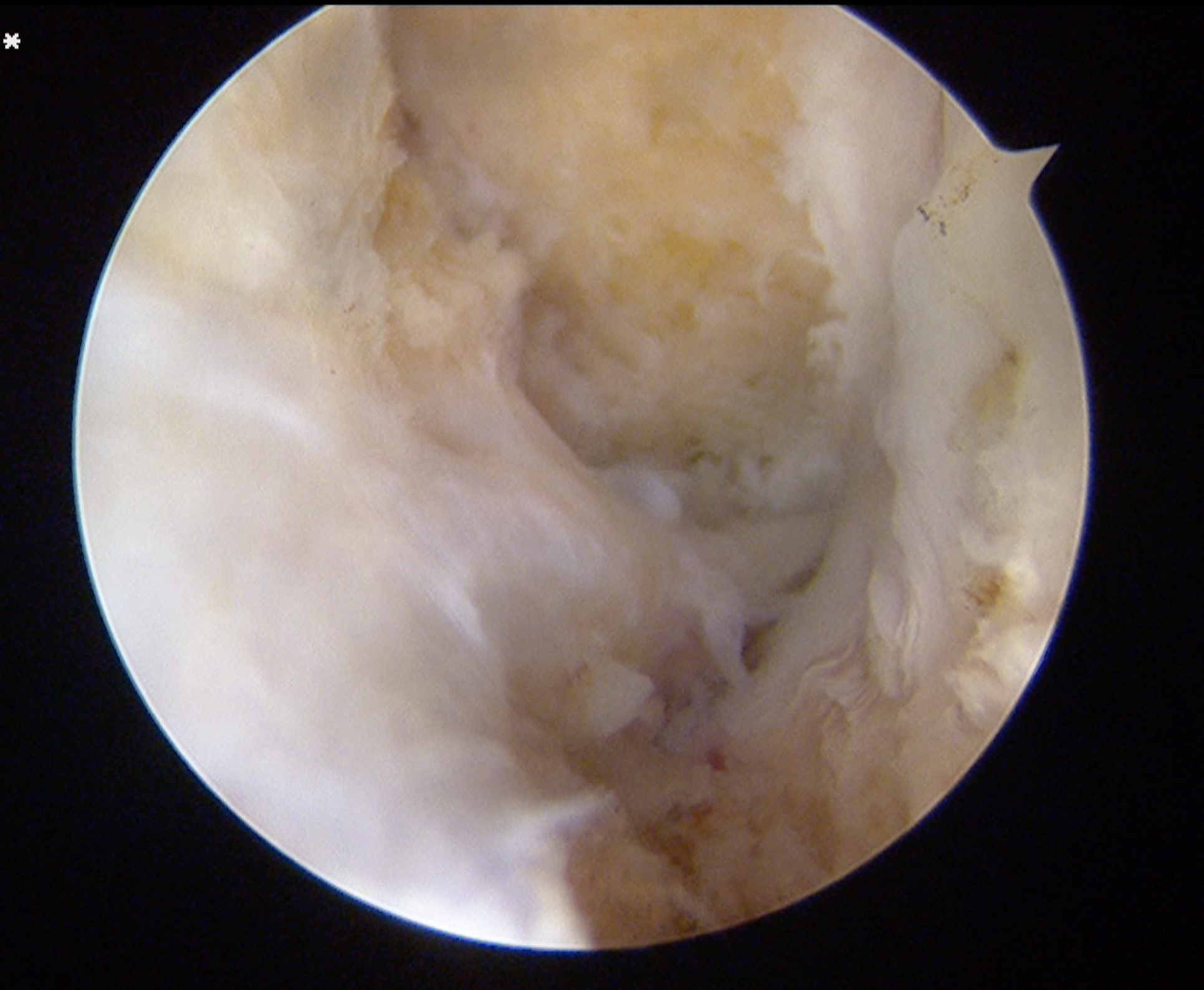

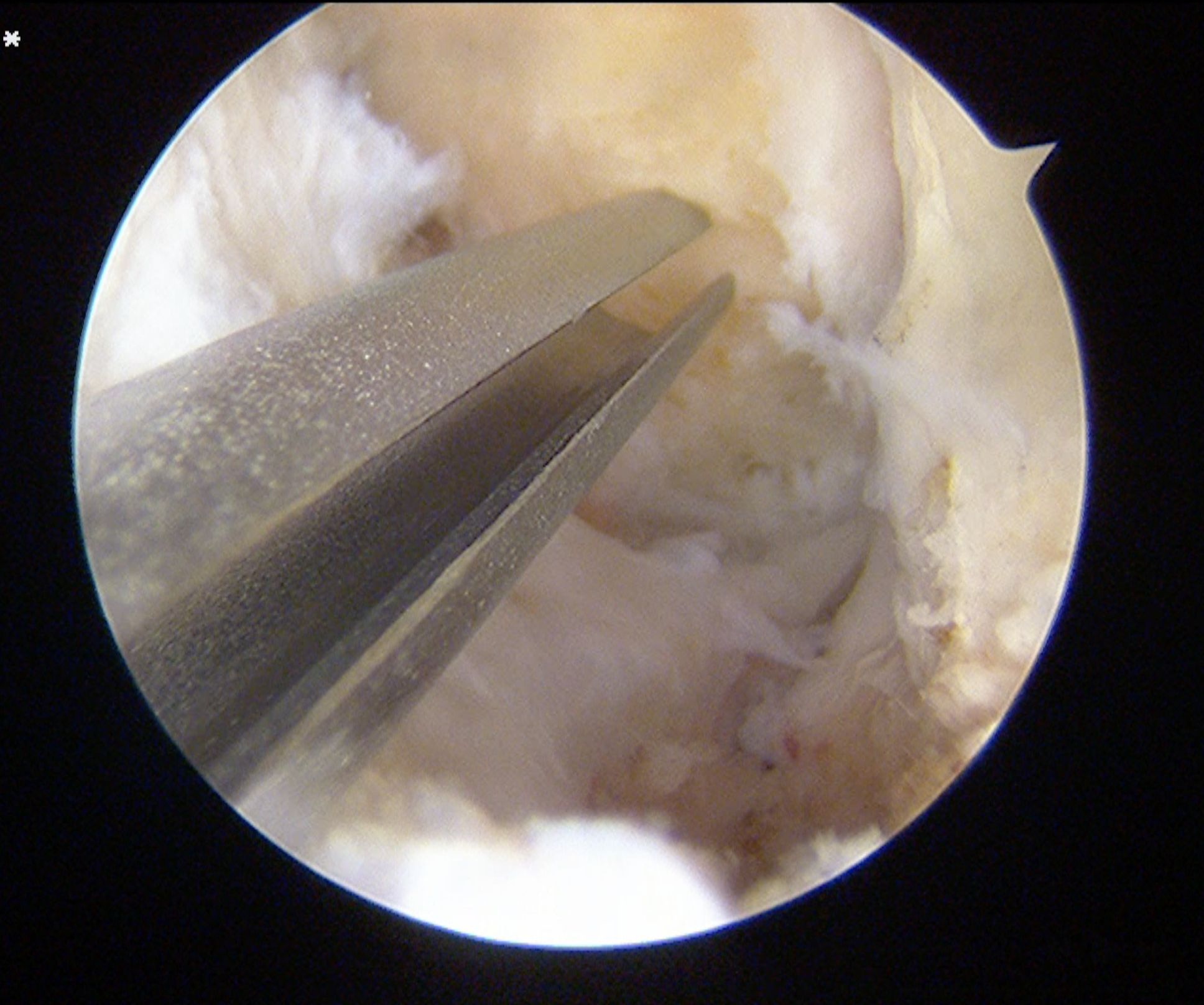

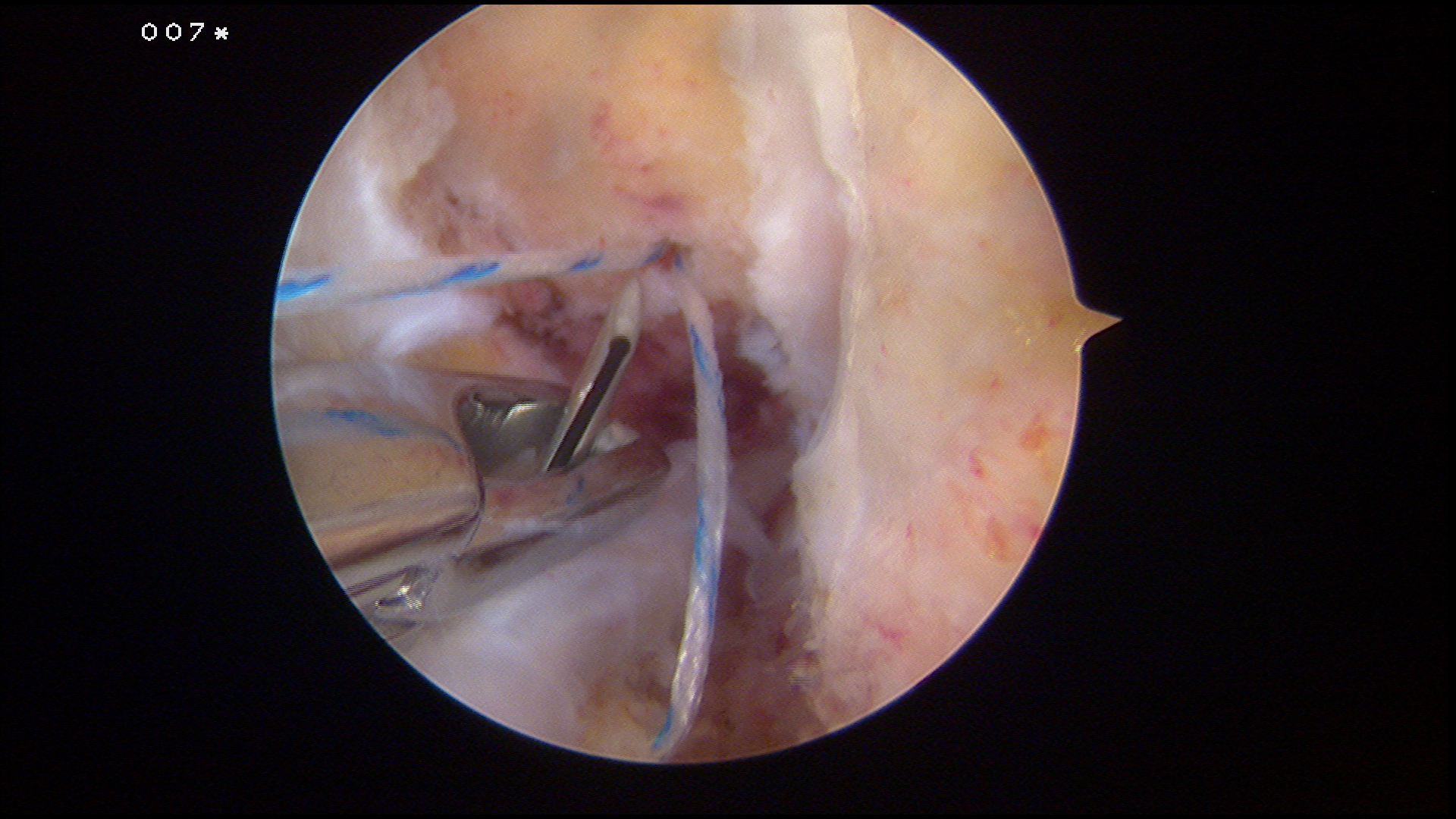


A

C

B

Figure S2. Arthroscopic ATFL repair: A. identification of the ATFL, B. drill guide for suture placement at the ATFL footprint, C. suture retriever for ATFL repair and tensioning of the ligament.


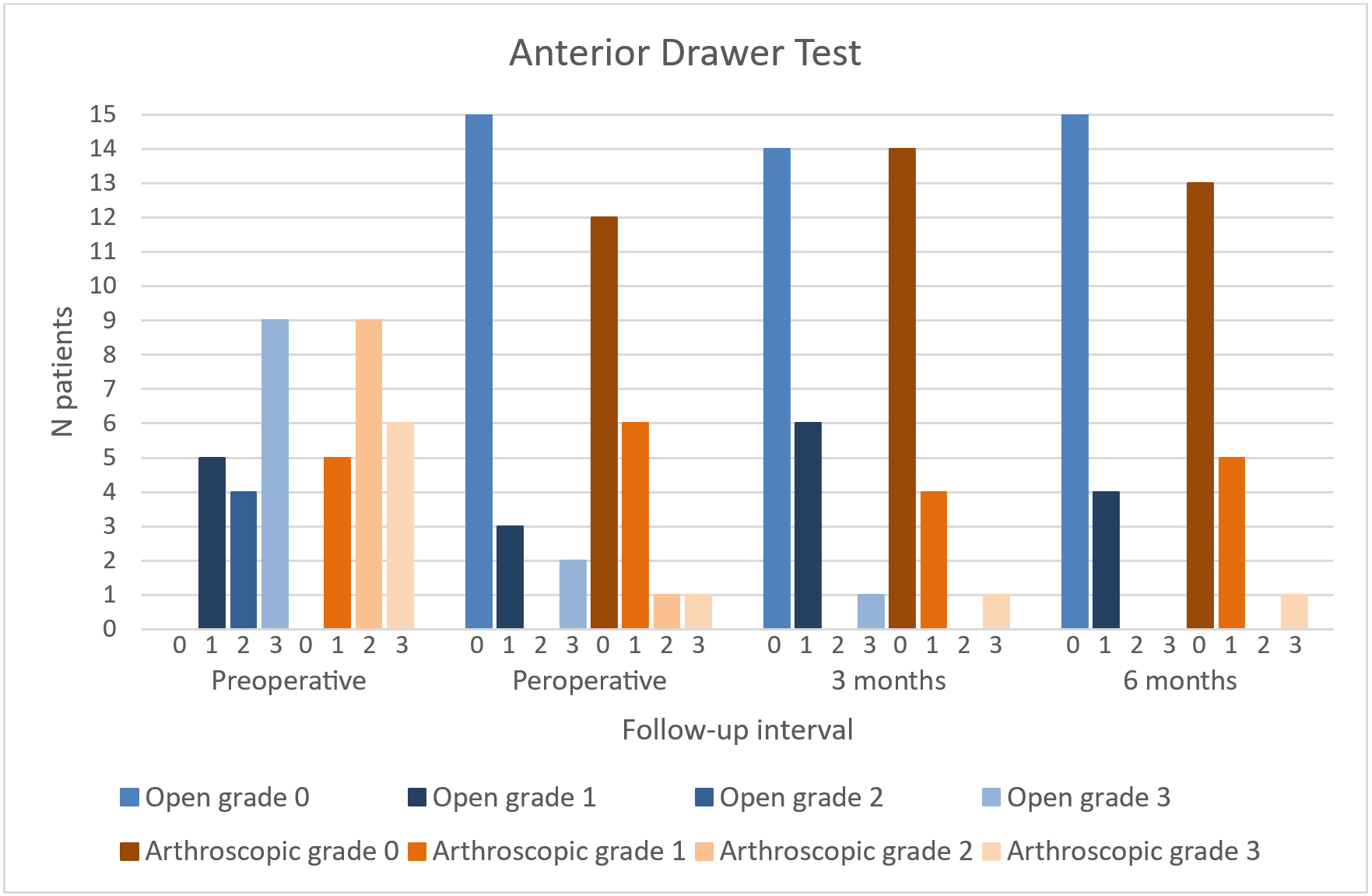


Figure S3. Quantitative overview of improvement in mechanical ankle instability as measured using the ADT per follow-up interval.
